# Mediation in Healthcare: Enhancing Conflict Resolution Between Patients and Physicians Beyond the Courtroom

**DOI:** 10.7759/cureus.75487

**Published:** 2024-12-10

**Authors:** Kostadin Dimitrov, Tsonka Miteva-Katrandzhieva

**Affiliations:** 1 Social Medicine and Public Health, Medical University of Plovdiv, Plovdiv, BGR

**Keywords:** alternative dispute resolution, clinical conflict management, healthcare conflict resolution, healthcare mediation, mediation, medical disputes, physician-patient communication, physician-patient mediation, physician-patient relationship

## Abstract

Healthcare is defined by rapidly advancing technologies and increased patient expectations, resulting in frequent disagreements between patients, their families, and medical practitioners. Historically, these conflicts have been settled through the adversarial court system, which frequently fails to produce equitable results due to unequal legal representation, procedural difficulties, and other shortcomings. This analysis investigates mediation, a type of Alternative Dispute Resolution (ADR), as a viable option for addressing healthcare disputes. Mediation is a voluntary, flexible, and confidential process in which a neutral third party organizes negotiations to help conflicting parties reach an agreement. Unlike litigation, mediation promotes open communication, empathy, and the preservation of relationships, which enhances patient trust and satisfaction. Furthermore, mediation resolves disputes more quickly and cost-effectively than traditional court proceedings, significantly reducing the emotional and financial burdens on all parties involved. This review examines the benefits of mediation, focusing on its role in preserving physician-patient relationships, reducing legal costs, and improving overall healthcare outcomes. The use of mediation in healthcare conflict resolution provides a more effective, compassionate, and lasting resolution that benefits healthcare institutions, practitioners, and patients.

## Introduction and background

Healthcare providers face significant challenges as a result of rapidly evolving technologies and increasing patient expectations. Disagreements between patients and/or their families and medical professionals occur frequently in daily practice [1]. Many authors consider conflicts in healthcare to be inevitable, underscoring the growing importance of this issue [2]. Without competent and timely management, disputes can have severe consequences on healthcare institutions, their staff members, and, especially, on patients [3].

Conflicts in healthcare are generally resolved through the legal system, which is inherently adversarial. During legal proceedings, both parties are required to define the issue in dispute and present supporting evidence to the court, upon which the judge issues a ruling in favor of one of the parties. However, the judicial process may not always result in equitable outcomes due to systemic limitations, including disparities in legal representation, insufficient or unreliable evidence and witnesses, and the potential for subjective bias [4]. From a healthcare perspective, the judicial system serves two purposes: compensation to those who suffer from medical errors and prevention of their occurrence. However, it is slow and expensive to apply in practice and tends to leave plaintiffs under-compensated in courtroom litigation [5]. The adversarial nature of the legal process additionally undermines the relationship between medical professionals and patients and damages patient trust in physicians generally [6]. The relationship between physician and patient is dynamic and complex but is also the key to optimal treatment [7].

Alternative Dispute Resolution (ADR) is a term that covers a wide range of approaches for resolving conflicts in a non-confrontational way, without the intervention of the court system [8,9]. One of the most significant and effective forms of ADR is mediation. Without the need for courts, a third party (the mediator) helps the conflicting parties reach a consensus through a voluntary, flexible, and private procedure [10]. The benefits of using mediation to resolve disputes in the healthcare sector include its flexible nature, the ability for parties to withdraw from mediation at any time, and the confidentiality of all information related to it. The parties are allowed to select the mediator. The mediation procedure may also be ended at the mediator's discretion or when one of the parties withdraws. Mediation helps to maintain good relations between the parties by ensuring the parties' commitment to follow the mediation agreement and offering a legal remedy if it is violated [11].

This review examines mediation as an alternative approach for resolving disputes between patients and physicians, contrasting it with the traditional legal system. While exploring how mediation gives parties the ability to actively search for mutually beneficial agreements out of court, this paper further evaluates how mediation can effectively improve the dispute resolution procedure in the healthcare environment by inducing a more empathetic and parties-centered dispute resolution strategy. This review addresses the growing need for conflict resolution methods that reduce the emotional, financial, and systemic burdens associated with legal disputes in healthcare.

## Review

Materials and methods

This literature review was conducted to analyze the advantages of mediation as a method of conflict resolution between patients and physicians and to compare it with the traditional court system. To accomplish this, a comprehensive study of peer-reviewed research articles was conducted in PubMed, Google Scholar, Web of Science, and Scopus databases. The reference lists of all included articles were reviewed to identify additional studies.

The following keywords were used: "mediation," "mediation in healthcare," "conflict in healthcare," "physician-patient conflict," "physician-patient relationship," and "alternative dispute resolution."

No specific time or geographic frame was set for the keyword searches, as the goal was to capture a broad range of studies on the topic. However, only articles published up until July 2024 were included in the review to ensure the inclusion of the most current research available at the time of the search. Although the majority of the articles incorporated are from the last 10 years, older studies were also included to provide a more comprehensive understanding of the topic, as they offer valuable and relevant insights. The inclusion criteria specifically focused on research papers, reports, and studies that addressed mediation, conflict resolution, and ADR between physicians and patients. Studies focusing on conflict resolution within healthcare teams or the use of mediation to facilitate healthcare access for vulnerable groups were excluded. Additionally, studies on mediation unrelated to conflict resolution in healthcare were not considered. The selection process involved screening titles and abstracts, followed by a full-text review of the eligible articles.

The literature search was conducted over a period of four months, from March 2024 to July 2024.

As this study is a review of existing literature, ethics approval was not required.

Conflicts in healthcare

Nature and Sources of Conflict

Conflict is a result of misunderstanding or failure to agree based on different positions, interests, or beliefs that two individuals or groups have, which later ends up resulting in an antagonistic state [12,13]. A significant basis of conflict within a healthcare environment is when the patient or their family members are not satisfied with the healthcare being provided to them [14].

Among the predisposing factors of conflict are higher expectations from patients, diversity of patients, and the presence of groups of patients who have difficulties with making decisions: elderly patients, young patients, patients of different ethnic groups, those with low education, differences in personal characteristics between patients and physicians, patients with specific health problems (infectious diseases, addictions) [1,15]. It's also a field that's prone to conflict, because it is complex and intricate, involving specialists and services of many kinds. It is also a personal activity that arouses strong emotions in both patients and health professionals [16].

Communication Issues as a Cause of Conflict

The most common cause of conflict between physicians and patients is poor communication and the quality of information provided by the medical professional to the patient [17,18]. Patients and their families are more likely to sue and get in conflict with physicians if they feel that adequate information about their condition and treatment was not given to them, if they feel neglected, and that their concerns were not adequately addressed [19]. According to studies, physicians who manifest higher levels of rudeness, poor communication with their patients, or are disrespectful, are at increased risk of conflicts with patients and are more likely to be sued [20-22]. Some patients are reluctant to ask further questions because they believe it would be seen as disrespectful or mistrustful of the medical practitioner, which exacerbates communication problems [23-26]. At the same time, studies show that a significant percentage of patients, between 15% and 60%, are perceived by healthcare professionals as "difficult," even though physicians are trained in active listening or effective communication techniques [27-30]. Poor communication compromises the quality of patient treatment. Effective medical care depends on a physician-patient relationship founded on trust, which enables information sharing during history collection, diagnostic procedures, and adherence to the treatment plan. Patients must trust their physician to fully benefit from their expertise and guidance [31, 32]. Additionally, patients perceive a decrease in the quality of healthcare when unresolved conflicts persist [33]. On the other hand, disagreements lead to higher stress levels in physicians, which are linked to higher rates of burnout, lower productivity, worse quality of life, and lower job satisfaction [34-36].

Consequences of Unmanaged Conflict 

Conflicts can have a negative impact on medical facilities (lawyer and court fees, increased employee turnover, and decreased productivity), staff (professional burnout, strained relationships with colleagues, and decreased well-being), and particularly on patients (medical error consequences, deteriorating physician-patient relationships, and compromised health outcomes) if they are not effectively and quickly managed [3].

Mediation: definition, principles, objectives

Mediation is a form of ADR distinguished by its voluntary, flexible, and confidential nature. This procedure assists disputing parties in reaching a resolution without judicial action, with the assistance of a third party (mediator) [10]. The mediator helps the parties identify their concerns, locate and explore their interests, and establish a safe, trusting atmosphere in which they can discuss their emotionally and psychologically complex issues. The mediator guides the conversations to resolve issues and reach a consensus. Unlike a judge or jury, the mediator does not render final decisions or take sides in the dispute [37,38]. In resolving conflicts, the mediator must not have a direct stake in the conflict or its outcome [8].

Principles of Mediation

Several fundamental principles guide mediation. Equality ensures that each party to the conflict has the same rights during the mediation process. The concept of voluntariness emphasizes that mediation is entirely voluntary; parties enter the process voluntarily and can leave at any time. The mediator maintains impartiality and neutrality, avoiding any bias or influence on the resolution of the conflict, and settlement occurs only by mutual agreement. Finally, confidentiality is crucial because all information revealed during mediation is kept strictly confidential and cannot be disclosed by the mediator or either party [39, 40].

Stages of the Mediation Process

The mediation process consists of several essential stages. Initially, the mediator sets up a voluntary meeting between the parties. During the first phase, the mediator introduces the mediation process and its principles to the participants. Next, the mediator investigates and clarifies the interests of both parties. During the negotiation and problem-solving phase, the mediator encourages each party to propose ideas that satisfy both parties. Once a mutually satisfactory resolution is achieved, the mediator prepares a formal agreement [41].

Objectives of Mediation

Mediation serves as a dispute resolution process designed to reach an effective, cost-efficient, and expedited consensus while also reducing court congestion. One of the goals of mediation is to eliminate obstacles such as economic, organizational, and procedural impediments that frequently prevent parties from accessing justice. In the mediation process, the parties determine the flow of the process, identify the needs and desires of each party, develop options, and reach agreements that are truly agreeable to all involved, ultimately moving beyond the dispute altogether. Furthermore, mediation promotes reform in how parties communicate and aims to alter social approaches to dispute resolution. It settles disagreements outside of the public domain while serving the interests of the parties concerned [42].

Mediation in healthcare

Adversarial Conflict Resolution in Healthcare: The Potential of Mediation

Physicians and patients are not naturally opposed; on the contrary, their relationship is built on the patient's vulnerability and trust, as well as the physician's expertise. Conflicts between medical professionals and patients could jeopardize this crucial relationship [43]. Traditionally, these issues are resolved through the legal system, which is fundamentally adversarial. In court disputes, both sides determine the source of the disagreement and present evidence for the judge to rule in favor of one side. However, the judicial system is not always capable of producing equitable results due to intrinsic defects such as unequal legal representation, flaws in the evidence presented, witness unreliability, and subjectivity [4]. Because of these flaws, many people who have been seriously injured as a result of medical errors are unable to obtain compensation. Furthermore, in cases where compensation is granted, the amount is often unpredictable [44]. Typically, in mediation, in contrast to a traditional court case, an agreement is reached only when both parties' needs are met. If this is not achieved, they reserve the right to discontinue the process and take the matter to court [43]. A trial requires the plaintiff to prove the defendant's guilt, and the judge must rule based on the evidence given, witness testimony, expert opinions, and current legislation. This process entails adherence to several procedures and rules, which can be daunting for the plaintiff. Expert witnesses play a crucial role in medical malpractice proceedings. Most physicians are hesitant to serve as expert witnesses, complicating the process even further. This reluctance can be attributed to a variety of factors, including an unwillingness to miss work due to a heavy workload, postponement of responsibilities that may not be in the best interests of their patients, time lost due to hearing delays, and a lack of understanding of legal procedures. Mediation, on the other hand, allows opposing parties to retain control of the dispute and reach a satisfactory conclusion without having to prove fault [45,46].

Cost of Traditional Litigation in Healthcare

The traditional court system in healthcare is burdened by substantial expenses associated with full-scale medical malpractice litigation, including compensation, legal fees for plaintiffs and defendants, defensive medicine costs, and other expenses. Legal expenses in the US healthcare system exceed $55 billion annually [47]. Despite the fact that 86% of medical malpractice actions against physicians in the United States conclude in a physician's favor, legal fees range from $24,000 to $90,000 per case. Only 1% of cases receive a verdict in favor of the plaintiff [48]. At the same time, in the United States, patients who receive court settlements for medical malpractice may be required to pay attorney fees of up to 30-40% [49]. Legal expenses for healthcare in the United Kingdom account for 0.04% of the country's GDP [4]. Furthermore, significant administrative and legal fees frequently deter many patients from filing cases. In Canada, the initial cost of filing a lawsuit can range between $40,000 and $50,000 [6]. When compared to full-scale court cases, mediation is more cost-effective since it requires less preparation time and frequently eliminates the need for attorneys [50]. Hertzler (2005) reported that using a collaborative, interest-based strategy to manage conflicts saved Children's Hospital in Atlanta approximately $52,000 per case [51]. Jury Verdict Research shows that resolving conflicts before they go to court saves an average of $50,000 in litigation costs per case. Additionally, attorney fees are greatly reduced. Lawyers indicated that the average preparation time for trials was 36 hours, compared to only 2.5 hours for mediation [9].

The Impact of Effective Communication Facilitated by a Mediator on Conflict Resolution in Healthcare

The general consensus is that patients file lawsuits primarily due to inadequate or interrupted communication with their physicians. This problem is exacerbated by patients' reluctance to ask additional questions, as well as doctors' avoidance of discussions with patients when treatment results are unexpected. Most lawsuits against physicians are filed not due to medical errors, but because of insufficient communication between patients and their physicians, as well as emotional tension on the patients' behalf [19,52,53]. The adversarial nature of the judicial system is also emotionally taxing for patients, making it difficult to obtain the most important aspects for them: an explanation of what happened, an apology from the physician, information on how similar situations will be avoided in the future, and compensation that satisfies the affected patient [54,55].

According to Galton, an American mediator specializing in healthcare dispute resolution, mediation provides patients with several advantages beyond the possibility of compensation: the opportunity to explain or receive an explanation (free communication); expression of regret; an apology; empathy; sympathy; the possibility of achieving case closure; granting forgiveness; and the restoration of a valuable relationship. Unlike litigation, mediation allows for an open and secure conversation with the help of a mediator, enabling the parties to listen actively and understand each other's needs and demands [54]. This leads to 90% satisfaction on both sides [9]. The mediation process is voluntary, confidential, and supportive, which promotes open communication and helps to maintain relationships and trust between patients and physicians. Furthermore, the informal setting encourages clinicians to communicate more openly with patients [1,6]. The clash between physician and patient in court harms their relationship and diminishes overall patient trust in medical professionals. Mediation, which is voluntary, confidential, and supportive, encourages open communication and can help to maintain trust and relationships. Its informal setting enables clinicians to be more honest with patients [1, 6]. Unlike judicial proceedings, mediation seeks to resolve disagreements and rebuild relationships between parties [56]. The physician-patient relationship is complex and dynamic, yet it is quite essential for effective treatment [7].

Defensive Medicine

The major function of the judicial system is to ensure that healthcare professionals provide high-quality, standard-compliant services. However, fear of punishment is ineffective at preventing medical errors or increasing healthcare system productivity. Instead, open communication mechanisms for discussing system flaws and implementing corrective solutions are critical for lowering medical errors and strengthening the entire healthcare system [57]. Furthermore, physicians are more inclined to make essential changes after reaching an agreement rather than after a public and humiliating court trial [58]. According to the United States House of Representatives, just 3% of those who have been hurt by medical errors file a lawsuit, while 80% of medical error claims do not involve genuine harm. These findings suggest that the court system neither properly compensates those harmed by medical errors nor efficiently controls the advancement of healthcare [48]. This statement is supported by data from Italy, where 80% of lawsuits against physicians are dismissed. Nonetheless, these professionals face the stress of lengthy and taxing legal battles, as well as reputational damage [27]. Excessive judicial suppression may have unanticipated effects [59].

One example is the practice of defensive medicine, which involves physicians deviating from standard medical practice out of fear of lawsuits [60]. Defensive medicine is characterized as performing numerous diagnostic and therapeutic procedures with the goal of protecting physicians from legal action rather than improving patient health [27]. This approach has several negative consequences for the quality of medical care, including providing inefficient or even potentially dangerous treatment, denying beneficial treatments, and generating excessive healthcare costs. Furthermore, defensive medicine damages the physician-patient relationship by shifting the focus from the patient's best interests to the physician's own protection [61]. In a survey of specialties with high litigation risks, 93% of physicians acknowledged practicing defensive medicine occasionally or regularly, with 42% reducing their clinical activities for fear of lawsuits. Furthermore, 59% admitted to performing unnecessary tests and treatments, while 39% avoided dealing with high-risk patients [62]. A survey of 300 general practitioners discovered that fear of legal action has resulted in significant changes in practice. 63.8% reported more referrals, 63.4% noted more follow-up visits, 59.6% observed increased diagnostic tests, 41.9% avoided treating certain conditions in primary care, 29.3% prescribed unnecessary treatments, and 25% removed patients from their list [63]. In a national study of U.S. physicians on defensive medicine, 91% felt that excessive testing is frequently performed out of fear of lawsuits [64]. According to a survey conducted by the Catholic University of Milan, 77.9% of physicians engaged in defensive medicine in the month prior to the interview. In Italy, 82.8% of medical records contained unnecessary information, 69.8% of patients were admitted to hospitals without a reason, 61.3% ordered excessive diagnostic tests, 58.6% sought unnecessary specialist consultations, 51.5% prescribed unnecessary medications, and 26.2% excluded high-risk patients from possible treatments. The main reasons for these activities were fear of legal disputes (80.4%), previous legal experiences (65.7%), concerns about compensation claims (59.8%), personal legal experiences (51.8%), and fear of negative media coverage (43.5%) [27]. Defensive medicine raises healthcare costs, increases risks, and creates emotional strain on patients, particularly with invasive procedures [62]. Additionally, it reduces access to healthcare since physicians may avoid procedures with high risks out of fear of lawsuits [65]. In the United States, hospital costs associated with defensive medicine account for 5.4% of total expenses, with defensive medicine costs totaling $45.6 billion in 2008 and averaging between $83 and $151 billion per year. These expenditures could be minimized by reforming the judicial system [9, 47].

Time to Resolution

One significant disadvantage of the traditional court system is the time required to receive a decision and conclude a case. Delays can occur at any stage of the court process, including obtaining legal counsel, gathering information from the opposing party, generating expert reports, exchanging documentary evidence, and scheduling trials [4]. The United States General Accounting Office reports that it takes an average of 14 months to file a lawsuit against a physician, while Jury Verdict Research found that resolving a medical malpractice case took an average of 45 months in 2000 [44]. In Canada, the time from filing a claim to reaching court is 5-6 years [6]. In Malaysia, the judicial process for medical malpractice cases takes a minimum of 15 years and can extend up to 25 years [44].

In contrast, a survey of the thirteen largest mediation firms in the U.S. found that the average time to reach a consensus in mediation is 1 to 3 days [9]. A study conducted in New York found that for 24 medical malpractice cases referred to mediation, the average time to reach a settlement was 2.34 hours, and the preparation time for attorneys was ten times shorter compared to preparation for court proceedings in the same cases [66]. Another study found that the time it takes to reach a settlement in mediation ranges from a few hours to several months in more formal disputes, with 90% of cases fulfilling the parties' expectations. Furthermore, legal costs are greatly decreased, with mediation requiring an average preparation time of 2.5 hours against 36 hours for court cases [51]. Furthermore, the quick resolution of conflicts through mediation reduces emotional stress for both parties, allowing physicians to continue their clinical practice without the burden of protracted litigation [1].

Discussion

This review underlines the benefits of mediation as a conflict resolution method between patients and physicians, comparing it with the traditional court system. It also highlights several limitations within the legal system in healthcare conflict resolution, including unequal legal representation, issues with evidence reliability, witness credibility, and subjectivity. These limitations can lead to situations where seriously injured patients, affected by medical errors, fail to receive fair compensation [4, 44].

Court cases also tend to be prohibitively expensive, with legal fees in the United States reaching up to $90,000 per case [48], while the United Kingdom allocates approximately 0.04% of its GDP to legal expenses in healthcare [4]. These financial resources could be more effectively redirected to improve healthcare services. Additionally, the looming threat of lawsuits often compels physicians to practice defensive medicine, which may compromise patient care. Studies reveal that as many as 90% of physicians engage in defensive practices due to litigation concerns [62]. Another significant drawback of the conventional legal system is the extended timeframe for resolving cases, which can span up to 25 years in some regions, such as Malaysia [44]. The adversarial nature of lawsuits also tends to overlook the root cause of many malpractice claims-a breakdown in communication between patients and physicians [19, 52, 53].

Mediation, in contrast, encourages open dialogue and aims to preserve the essential trust between patients and physicians, leading to a 90% satisfaction rate among both parties involved [1, 6, 7, 9, 54]. The cost savings from each case resolved through mediation can reach up to $52,000 [51], freeing funds that can be allocated to other critical healthcare needs. Moreover, mediation often achieves resolution within a few hours, a stark contrast to the prolonged duration of traditional litigation [66].

Mediation presents a compelling alternative to litigation by promoting timely, cost-effective, and trust-preserving resolutions. Shifting towards mediation can reduce the financial burden on healthcare systems and improve the overall quality of patient care. Embracing this approach may pave the way for a more effective and harmonious method of addressing medical disputes.

Limitations

As this literature review is not geographically restricted and aims for broad comprehension, it includes studies from various countries with differing legal costs. Moreover, each country has its unique cultural context, which may influence the success rates of mediation as an alternative method for conflict resolution between patients and physicians. Therefore, the legal expenditures saved through mediation and the success rates of this procedure reported in this study may not be directly applicable or transferable to different regions. Despite its limitations, this study highlights the advantages of mediation compared to the traditional court system, making it a valuable source of information, especially for countries that have not yet implemented this method, and encouraging further investigation.

## Conclusions

The adversarial nature of the traditional judicial system, along with its lengthy resolution timelines and high legal fees, frequently undermines the effectiveness of this approach. This adversely affects healthcare institutions, providers, and patients, leading to heightened stress and burnout among healthcare professionals and potentially worsening patient satisfaction. Mediation, as a voluntary, flexible, and confidential alternative, not only resolves disagreements more quickly and affordably but also reduces the psychological strain on all parties involved and helps to maintain and reestablish the vital physician-patient relationship. In conclusion, the settlement process can be considerably enhanced by incorporating mediation into healthcare dispute resolution systems.
